# Supplementation of Dimethylglycine Sodium Salt in Sow Milk Reverses Skeletal Muscle Redox Status Imbalance and Mitochondrial Dysfunction of Intrauterine Growth Restriction Newborns

**DOI:** 10.3390/antiox11081550

**Published:** 2022-08-10

**Authors:** Kaiwen Bai, Luyi Jiang, Qiming Li, Jingfei Zhang, Lili Zhang, Tian Wang

**Affiliations:** 1 School of Biological and Chemical Engineering, Zhejiang University of Science and Technology, Hangzhou 310023, China; 2 College of Animal Science and Technology, Nanjing Agricultural University, Nanjing 210095, China; 3 Ministry of Education Key Laboratory of Molecular Animal Nutrition, Institute of Dairy Science, College of Animal Sciences, Zhejiang University, Hangzhou 310058, China

**Keywords:** intrauterine growth restriction, skeletal muscle, redox status, mitochondrial dysfunction, dimethylglycine sodium salt

## Abstract

The current study sought to understand the mechanism underlying skeletal muscle dysfunction brought on by intrauterine growth restriction (IUGR) and to explore the treatment benefits of applying dimethylglycine sodium salt (DMG-Na) in sow milk to newborns during the suckling period. Each of the 10 sows delivered one newborn with a normal birth weight (NBW) and one with an IUGR. Additionally, two NBW and two IUGR newborns were collected per litter of another 10 sows. The 20 NBW newborns were divided between the N (sow milk) and ND (sow milk + 0.1% DMG-Na) groups, while 20 IUGR newborns were divided between the I (sow milk) and ID (sow milk + 0.1% DMG-Na) groups. The skeletal muscle histomorphology, redox status, and levels of gene and protein expression were worse (*p* < 0.05) in the I group than in the N group. In addition, supplementation with DMG-Na (ND and ID groups) improved (*p* < 0.05) those parameters compared to the unsupplemented groups (N and I groups). Inhibited nuclear factor erythroid 2-related factor 2 (Nrf2)/sirtuin 1 (SIRT1)/peroxisome proliferator-activated receptorγcoactivator-1α (PGC-1α) activity resulted in decreased redox status, skeletal muscle structural damage, skeletal muscle mitochondrial function impairment, and decreased performance in IUGR newborns. Supplementation of DMG-Na in sow milk activated the Nrf2/SIRT1/PGC-1α in IUGR newborns, thereby improving their skeletal muscle performance.

## 1. Introduction

A significant issue in human medicine is intrauterine growth restriction (IUGR), which is seen in fetuses who weigh less than the 10th percentile or the population mean less than two standard deviations of a population-based nomogram [1]. Postnatal development, nutrition utilization efficiency, and long-term health are all permanently stunted by IUGR [2]. This is due to poor food intake, disease, or oxidative damage caused by neonates with IUGR. Growth limitation and metabolic diseases are linked to dysfunctional skeletal muscles. Skeletal muscle is moreover particularly vulnerable to nutritional deficiency in pregnancy because it has a low priority for nutrient partitioning [3]. Therefore, in neonates with IUGR, slow skeletal muscle growth after birth may contribute to slower postnatal growth rates, and the skeletal muscle’s altered redox status and mitochondrial function may have a negative effect on the performance of newborns with IUGR across the whole postnatal development and subsequent in adulthood [4].

Since mitochondria are the primary sources of energy for the cell and use cellular respiration to turn nutrients into energy, changes to their structure and function are linked to a variety of diseases [5]. The activity of the mitochondrial respiratory chain is regulated by nuclear factor erythroid 2-related factor 2 (Nrf2), which is also involved in activating the antioxidant network [6]. A coactivator with a significant pleiotropic role in mitochondrial biogenesis is peroxisome proliferator-activated receptor-γ coactivator-1α (PGC-1α), which promotes the expression of both nuclear and mitochondrial genes that encode mitochondrial proteins. Both enzymatic and non-enzymatic antioxidant defenses are activated as a result of the downregulation of its activity followed by an increase in reactive oxygen species (ROS) brought on by oxidative damage [7]. Sirtuin 1 (SIRT1), which was first identified as the factor controlling apoptosis and DNA repair, is known to control genomic stability and cellular metabolism and is extremely sensitive to the redox status of the cell [8]. Previous research has indicated that SIRT1 interacts physically with PGC-1α and deacetylates it at several lysine positions, inducing PGC-1α activity and affecting the redox status [9].

Dimethylglycine sodium salt (DMG-Na) is similar to choline and betaine, which are crucial for the production of glutathione, and can thus help the body maintain a redox status and alleviate oxidative stress by eliminating excessive reactive oxygen species. Previous research suggested that DMG-Na might enhance oxygen use, defending the organism against oxidative damage. Additionally, it improves the body’s immunological responses, therefore improving their bodies’ performance properly [10,11,12]. In this work, decreased SIRT1 activity through its substrate PGC-1α impacted the skeletal muscle’s redox status and mitochondrial function, lowering the performance of IUGR newborns. We also provide a new perspective on the role of the Nrf2/SIRT1/PGC-1α network in the effects of DMG-Na on the redox status and mitochondrial function of skeletal muscle in newborns with IUGR.

## 2. Materials and Methods

The experimental protocols for animal care were authorized by the Nanjing Agricultural University Institutional Animal Care and Use Committee (protocol number 00494SYXK(SU)2017-0027) and this trial was carried out in conformity with Chinese regulations for the welfare of animals.

### 2.1. Experiment Design

Twenty sows [Duroc × (Landrace × Yorkshire)] were used in this study, and 30 newborns with normal birth weight (NBW) (1.53 ± 0.04 kg) and 30 newborns with IUGR (0.76 ± 0.06 kg) were chosen using a previously reported method [13]. All sows were fed the same gestation and lactation diet that satisfied the National Research Council’s dietary requirements (NRC, 2012), and they all had the same birth order (Appendix A Table A1). A total of 10 NBW newborns (NBW group, 1.54 ± 0.07 kg) and 10 IUGR newborns (IUGR group, 0.74 ± 0.07 kg) were collected, representing one NBW and one IUGR newborn from each litter of 10 sows. Additionally, two NBW and two IUGR newborns were obtained from each litter of another 10 sows. Twenty NBW newborns were divided into the N (basic sow milk diet) and ND (basic sow milk diet + 0.1% DMG-Na) groups, and 20 IUGR newborns were divided into the I (basic sow milk diet) and ID (basic sow milk diet + 0.1% DMG-Na) groups. Two NBW newborns from one litter were divided into N and ND groups, and two IUGR newborns from one litter were divided into I and ID groups. From day 7 to day 21, the newborns received a standard sow milk diet in separate bottles. Additionally, they received 25 mg of ferrous sulfate (dissolved in 2 mL of normal saline) orally on the 2nd, 7th, 12th, and 17th days, respectively. The supplier of DMG-Na (99.9% pure) was Qilu Sheng Hua Pharmaceutical Co., Ltd. of Zibo, Shandong, China.

At ages 0, 7, 10, 13, 16, and 19, newborns were weighed. Every 2–3 h (about 9–10 times per day), warm milk was taken from the appropriate mother and given to the newborns. An experienced staff collected milk and calculated the body weight of each sow. Before the trial, the corresponding staff touched the sows gently every day to adapt the sows to the emergence of staff and reduce unnecessary stress response. In this study, we fed the newborns separately with bottles for 14 days in an effort to mimic the normal feeding circumstances for the newborns. The newborns in the four groups (N, ND, I, and ID) were nurtured in plastic houses (1.5 m × 0.7 m × 0.7 m) in a controlled environment (33 °C) with free access to water.

### 2.2. Sample Collection

Within two hours of birth, without suckling, the selected 10 NBW newborns and 10 IUGR newborns were electrocuted and slaughtered via jugular bloodletting. At an age of 21 days, 40 newborns from the N, ND, I, and ID groups (10 newborns per group) were anesthetized via electrical stunning and slaughtered by exsanguination, and their skeletal muscle samples [(forelimb muscles (FM), hindlimb muscles (HM), and longissimus dorsi muscle (LM)] were obtained. The skeletal muscle tissue was removed from the abdominal cavity immediately after death and used for further analysis.

### 2.3. Histomorphological Study

Skeletal muscle samples (1 cm × 1 cm × 1 cm) treated in 4% buffered formaldehyde were dried using a graded sequence of xylene and ethanol, then the samples were embedded in paraffin for histological analysis. Xylene was used to deparaffinize the samples (8 μm in size), and then graded ethanol dilutions were used to rehydrate them. Slides were incubated with eosin and hematoxylin (HE). An optical binocular microscope was used to collect the pictures from the ten slides that were prepared for each sample (middle site of samples). Additionally, the fibers’ cross-sectional area was calculated. Briefly, from each of the muscle samples, one of the 20 transversal sections was chosen at random. Then, a grid with compartments of 12 × 8 was used to partition the portion. Five sample fields were randomly chosen from this grid, and five fibers were randomly chosen and measured from each of these sample fields. A light microscope (Olympus BH-2) and morphological measuring software (Sigma ScanPro 4; Aspire Software International, Danvers, MA, USA) were used to examine a total of 25 fibers each muscle, that is, 150 fibers per group. Each muscle within each set of muscles had its mean cross-sectional area of the 25 fibers estimated, and a grand mean was established for comparisons between groups.

The LM samples were fixed for 7 days in 10% neutral buffered formalin, dehydrated in a gradient of 70–100% ethyl alcohol, washed in xylene, and then embedded in paraffin. The slices of the paraffin-embedded LM samples were 5 µm thick. Van Gieson’s (VG) staining was used to detect morphological changes lasting 2 min at room temperature. Under a light microscope, pathological alterations were seen. To accurately reflect the muscle fibrosis index, the area percentage of collagen fiber/muscle fiber was determined using stereological system image analysis software.

### 2.4. Redox Status Study

Two grams of skeletal muscle were homogenized in eight milliliters of 0.9% sodium chloride solution on ice before being centrifuged at 3500× *g* for 15 min at 4 °C. Using the appropriate assay kit and following the manufacturer’s instructions, the supernatant of skeletal muscles homogenate was used to measure the levels of superoxide dismutase (SOD), glutathione peroxidase (GSH-Px), glutathione, glutathione reductase (GR), catalase (CAT), and malondialdehyde (MDA) (Nanjing Jiancheng Institute of Bioengineering, Nanjing, Jiangsu, China). Using a bicinchoninic acid (BCA) protein test kit as directed by the manufacturer, the protein content was measured (Nanjing Jiancheng Institute of Bioengineering).

### 2.5. Mitochondria Redox Status Study

Using a mitochondria isolation kit, the mitochondria of the skeletal muscle samples were separated (Solarbio, Beijing, China). Using the appropriate test kit and following the manufacturer’s instructions, the levels of manganese superoxide dismutase (MnSOD), GSH-Px, GSH, GR, and -γ-glutamylcysteine ligase (γ-GCL) in the mitochondria of skeletal muscle were determined (Nanjing Jiancheng Institute of Bioengineering).

### 2.6. Oxidative Damage Study

A ROS assay kit was used to measure the ROS level (Nanjing Jiancheng Institute of Bioengineering). In a brief, the mitochondria were treated for 30 min at 37 °C with 10 M dichlorodihydrofluorescein diacetate (DCFH-DA) and 10 mmol/L DNA stain Hoechst 33342. In addition, use a fluorescence reader, the DCFH fluorescence of the mitochondria was detected at 530 nm for emission and 485 nm for excitation (FACS Aria III; BD Biosciences, Franklin Lakes, NJ, USA). The outcomes are shown as the mean DCFH-DA fluorescence intensity over that of the control. Using a mitochondrial membrane potential (MMP) test kit, MMP level was estimated (Nanjing Jiancheng Institute of Bioengineering). In a summary, the mitochondria were loaded with 1 × JC-1 dye at 37 °C for 20 min, washed, and then flow cytometrically evaluated (FACS Aria III). Increases in the green to red fluorescence ratio were used to assess MMP. The outcome was determined as the ratio of the fluorescence of aggregates (red) to that of the monomers (green).

Using an Alexa Fluor^®^ 488 Annexin V/Dead Cell Apoptosis kit, the quantity of necrotic and apoptotic cells was counted (Thermo Fisher Scientific, Inc., Waltham, MA, USA). To use a glass homogenizer, the skeletal muscle samples were pulverized; the cells were then washed twice with cold PBS buffer (pH = 7.4); and finally, they were resuspended (2% suspension) in 1 × Annexin binding solution. After calculating the cell density, 1 × Annexin binding buffer was used to dilute the cells to 1 × 10^6^ cells/mL. An adequate amount of the aforementioned cell culture was stained in the dark for 15 min using a staining solution containing an-nexin V-fluorescein isothiocyanate and propidium iodide (1:9). After incubation, the forward scatter of the cells was determined, and the intensity of An-nexin V-fluorescein was quantified in FL-1 using a FACS Caliber at an excitation wavelength of 488 nm and an emission wavelength of 530 nm (BD Biosciences). Following the manufacturer’s instructions, the ELISA assay kits were used to determine the protein carbonyls (PC) and 8-hydroxy-2-deoxyguanosine (8-OHdG) in the skeletal muscle samples (Nanjing Jiancheng Institute of Bioengineering).

### 2.7. Mitochondrial Electron Transport Chain Complexes Study

Using an ELISA kit according to the manufacturer’s instructions, the activities of the electron transport chain (ETC) complexes I, II, III, IV, and V in the skeletal muscle samples were determined (Nanjing Jiancheng Institute of Bioengineering).

### 2.8. Energy Metabolism Study

Using a commercial kit, the concentration of glycogen in the skeletal muscle samples was determined (Nanjing Jiancheng Institute of Bioengineering). NAD^+^ and NADH concentrations in skeletal muscle samples were measured using commercial kits in accordance with the manufacturer’s instructions (SinoBestBio, Shanghai, China). Following the directions provided by the manufacturer, an ATP assay kit (Solarbio, Beijing, China) was used to measure the amount of ATP contained in the skeletal muscle samples.

Using the ubiquitous Genomic DNA Extraction Kit, the complete genomic DNA of samples of skeletal muscle was extracted (TakaRa Biotechnology Co., Dalian, China). A spectrophotometer (NanoDrop 2000c; Thermo Scientific, Camden, NJ, USA) was used to measure the DNA concentration, and it was then diluted to the same concentration for subsequent real-time PCR analysis. By using real-time PCR analysis, the relative mitochondrial DNA (mtDNA) concentration was quantified. In a word, the 20 µL PCR combination contained 10 µL of SYBR Premix Ex Taq (2X), 0.4 µL of downstream primer, 0.4 µL of upstream primer, 0.4 µL of ROX dye (50X), 6.8 µL of ultra-pure water, and 2 µL of cDNA template. The mitochondrial D-loop gene’s upstream and downstream primer sequences were 5′-AGGACTACGGCTTGAAAAGC-3′ and 5′-CATCTTGGCATCTTCAGTGCC-3′, respectively; the β-actin upstream and downstream primer sequences were 5′-TTCTTGGGTATGGAGTCCTG-3′ and 5′-TAGAAGCATTTGCGGTGG-3′, respectively. Using β-actin as an internal standard and the result was determined using the 2^−ΔΔCt^ method [14].

### 2.9. Quantitative Real-Time PCR (qPCR) Study

qPCR was carried out as previously mentioned [14]. Following the manufacturer’s recommendations, total RNA was extracted from the LM sample using Trizol Reagent (TaKaRa, Dalian, China), and it was subsequently reverse-transcribed using a commercial kit (Perfect Real Time, SYBR@ PrimeScriptTM, TaKaRa). SYBR@ Premix Ex Taq TM II (Tli RNaseH Plus) and the ABI 7300 Fast Real-Time PCR detection equipment were used to measure the expression levels of genes using real-time PCR (Applied Biosystems, Foster City, CA, USA). A 0.4 μL of the forward and reverse primers, 0.4 μL of ROX reference dye (50X), 0.4 μL of ddH_2_O, 6.8 μL of SYBR^@^ Premix Ex Taq (2X), and 2 μL of cDNA template made up the SYBR Green PCR reaction mixture. Three duplicates of each sample were amplified. Using the *β-actin* gene as an internal standard, the 2^−ΔΔCt^ technique [14] was used to determine the fold-expression of each gene. In Appendix A Table A2, the primer sequences used are listed.

### 2.10. Western Blot Study

Utilizing lysis solution including a protease inhibitor cocktail for the radioimmunoprecipitation experiment, total protein was extracted from the LM samples in each group (Beyotime Institute of Biotechnology, Nanjing, China). Nuclear Protein Extraction Kit was used to extract the nuclear protein from the LM samples (Beyotime Institute of Biotechnology, Nanjing, China). Using a BCA protein assay kit, total cellular and nuclear protein concentrations in the LM samples were determined (Beyotime Institute of Biotechnology). We purchased antibodies against associated proteins from Cell Signaling Technology (Danvers, MA, USA). The same amount of protein was then separated using SDS-PAGE and transferred to polyvinylidene difluoride membranes. The membranes were then incubated for 1 h with blocking buffer (5% bovine serum albumin in Tris-buffered saline with 1% Tween 20), followed by priming with the primary antibodies (1:1000) against Nrf2 (# 12721S), heme oxygenase 1 (HO1; # 82206S), SOD (# 37385S), GSH-Px (# 3286S), SIRT1 (# 9475S), PGC-1α (# 2178S), occludin (OCLN; # 91131S), zonula occludens-1 (ZO1; # 13663S), Cytochrome C (Cyt C; # 11940S), mitochondrial transcription factor A (mtTFA; # 8076S), mitochondrial mitofusin 2 (Mfn2; # 9482S), dynamin-related protein 1 (Drp1; # 8570S), mitochondrial fission 1 (Fis1; # 84580S), and α-tubulin (# 2125S) overnight at 4 °C. The membranes were then treated with a suitable secondary antibody for 1 h after being rinsed with Tris-buffered saline containing 0.05% Tween 20. After autoradiography, enhanced chemiluminescence tools (ECL Kit, Beyotime, Nanjing, China) were used to identify the blots. Luminescent Image Analyzer LAS-4000 system (Fujifilm Co., Tokyo, Japan) was used to capture pictures of the membranes, and ImageJ 1.42 q was used to quantify the images (NIH, Bethesda, MD, USA).

### 2.11. Statistical Analysis

With group (G), time (T), DMG-Na (D), and G × T × D as fixed effects and newborns as random effects, a mixed model was used to predict the body weight data of repeated recordings during the suckling phase. The body weight of the various groups was compared using one-way ANOVA if the *p* value of the interaction G × T × D was less than 0.05. The data for the N, ND, I, and ID groups with various superscripts a, b, and c differed considerably (*p* < 0.05).

The data of redox status, oxidative damage, mitochondrial ETC, and energy metabolism among trial A (NBW, IUGR) and trial B (N, ND, I, ID) were examined independently. Using a mixed model with group (GA), skeletal muscle (MA), and GA × MA as fixed variables and newborns as random effects, the appropriate data for trial A were produced. For comparison between several groups in each segment of the skeletal muscles, paired *t*-tests were carried out if the *p* value of the interaction GA × MA was less than 0.05. A mixed model with group (GB), DMG-Na (D), skeletal muscle (MB), and GB × D, GB × MB, D × MB, GB × D × MB as fixed variables, and newborns as random effects, the appropriate data for trial B were generated. For comparison between several groups in each segment of the skeletal muscles, one-way ANOVA was carried out if the *p* value of the interaction GB × D × MB was less than 0.05.

Separate analyses were conducted on the information pertaining to the expression of the LM gene and protein in trials A and B. The newborns from the NBW and IUGR groups were paired with their litters for trial A, and the paired *t*-test was used to compare the two groups. Using a mixed model with group (GB), DMG-Na (D), and GB × D as fixed variables and newborns as random effects, the relevant data for trial B were produced. For the purpose of comparing several groups, one-way ANOVA was carried out if the *p* value for the interaction GB × D was less than 0.05.

There were significant differences between the values with various superscripts * (NBW, IUGR group) and a, b, c, d (N, ND, I, ID group) (*p* < 0.05). The Statistical Analysis System software (version 9.1; SAS Institute, Inc., Cary, NC, USA) was used to examine all of the data. The cutoff for statistical significance was *p* < 0.05. Data are shown as the mean with the standard deviation.

## 3. Results

### 3.1. Growth Performance

Table 1 illustrates the favorable benefits of DMG-Na on IUGR newborns’ growth during the suckling period. The interaction effects (groups × time × DMG-Na) were significant (*p* < 0.001) for bodyweight traits. The I group’s body weight values were lower than those of the N group’s (*p* < 0.05). Additionally, from day 16 to day 21 of the suckling period, the bodyweight values in the ND and ID groups improved (*p* < 0.05) in comparison to those in the N and I groups.

### 3.2. Histomorphological Study

**Figure 1:**
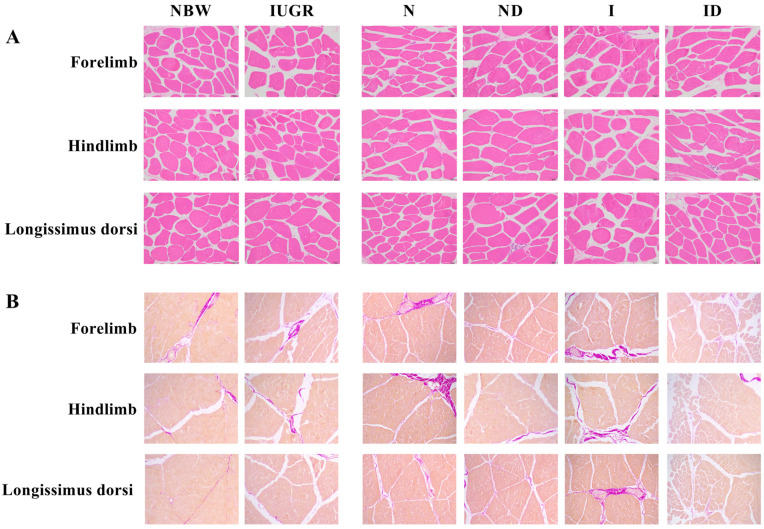
DMG-Na supplementation improved histomorphological characteristics of the skeletal muscles of IUGR newborns during the suckling period. (**A**) Hematoxylin-eosin staining, scale bars indicate 50 μm; (**B**) Van Gieson staining, scale bars indicate 50 μm. NBW, normal birth weight newborns; IUGR, intrauterine growth restriction newborns; N, NBW newborns are administered a sow milk diet; ND, NBW newborns are administered a sow milk diet + 0.1% DMG-Na; I, IUGR newborns are administered a sow milk diet; ID, IUGR newborns are administered a sow milk diet + 0.1% DMG-Na.

**Figure 2:**
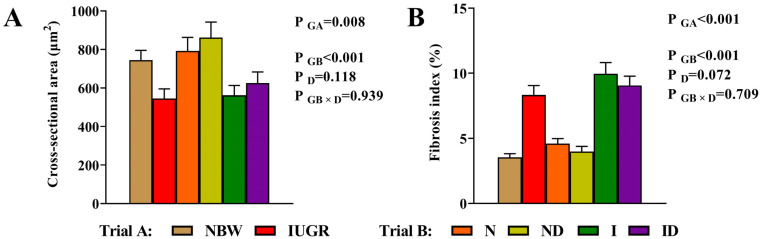
DMG-Na supplementation improved cross-sectional area and fibrosis index values of the skeletal muscles of IUGR newborns during the suckling period. (**A**) Cross-sectional area and (**B**) fibrosis index values of the skeletal muscles. Data are shown as the mean ± standard deviation, n = 10 newborns per group. Different *p*-values lower than 0.05 represent significant differences. NBW, normal birth weight newborns; IUGR, intrauterine growth restriction newborns; N, NBW newborns are administered a sow milk diet; ND, NBW newborns are administered a sow milk diet + 0.1% DMG-Na; I, IUGR newborns are administered a sow milk diet; ID, IUGR newborns are administered a sow milk diet + 0.1% DMG-Na.

The beneficial effects of DMG-Na on histomorphology of the skeletal muscles of IUGR newborns during the suckling period are shown in Figure 1 and Figure 2. In trial A, In comparison to the NBW group, the skeletal muscles of the IUGR group had a lower (*p* < 0.05) cross-sectional area of the fibers and a higher (*p* < 0.05) fibrosis index, making them more vulnerable to internal structural damage. In trial B, the interaction effects (group × DMG-Na) were not significant (*p* > 0.05) for any of the tested histomorphological traits. However, supplementation with DMG-Na ameliorated internal structure damage of the skeletal muscles in the ID group compared to the I group.

### 3.3. Redox Status Study

**Figure 3:**
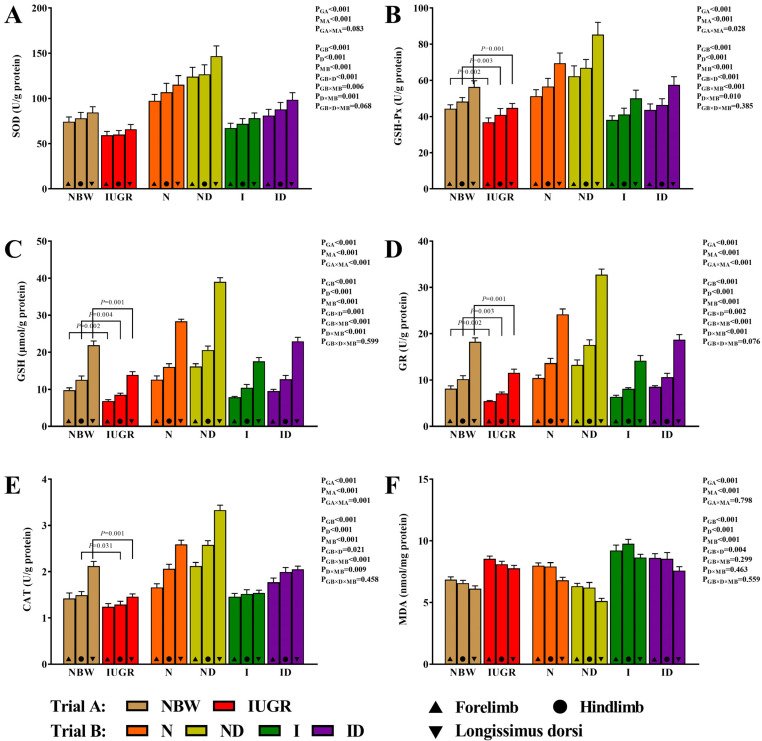
DMG-Na supplementation improved the redox status of the skeletal muscles of IUGR newborns during the suckling period. (**A**) SOD, (**B**) GSH-Px, (**C**) GSH, (**D**) GR, (**E**) CAT, and (**F**) MDA values of the skeletal muscles. Data are shown as the mean ± standard deviation, n = 10 newborns per group. Different *p*-values lower than 0.05 represent significant differences. NBW, normal birth weight newborns; IUGR, intrauterine growth restriction newborns; N, NBW newborns are administered a sow milk diet; ND, NBW newborns are administered a sow milk diet + 0.1% DMG-Na; I, IUGR newborns are administered a sow milk diet; ID, IUGR newborns are administered a sow milk diet + 0.1% DMG-Na. SOD, superoxide dismutase; GSH-Px, glutathione peroxidase; GSH, glutathione; GR, glutathione reductase; CAT, catalase; MDA, malondialdehyde.

Figure 3 shows the positive effects of DMG-Na on the redox state of the skeletal muscles of newborns with IUGR during the suckling period. In trial A, the interaction effects (group × muscle) were significant for the GSH-Px (*p* = 0.028), GSH (*p* < 0.001), GR (*p* < 0.001), and CAT (*p* = 0.001) traits. In comparison to the NBW group, the GSH-Px, GSH, GR, and CAT levels in the IUGR group were decreased (*p* < 0.05). For all of the evaluated redox status traits in trial B, the interaction effects (group × DMG-Na × muscle) were not significant (*p* > 0.05).

### 3.4. Mitochondrial Redox Status Study

**Figure 4:**
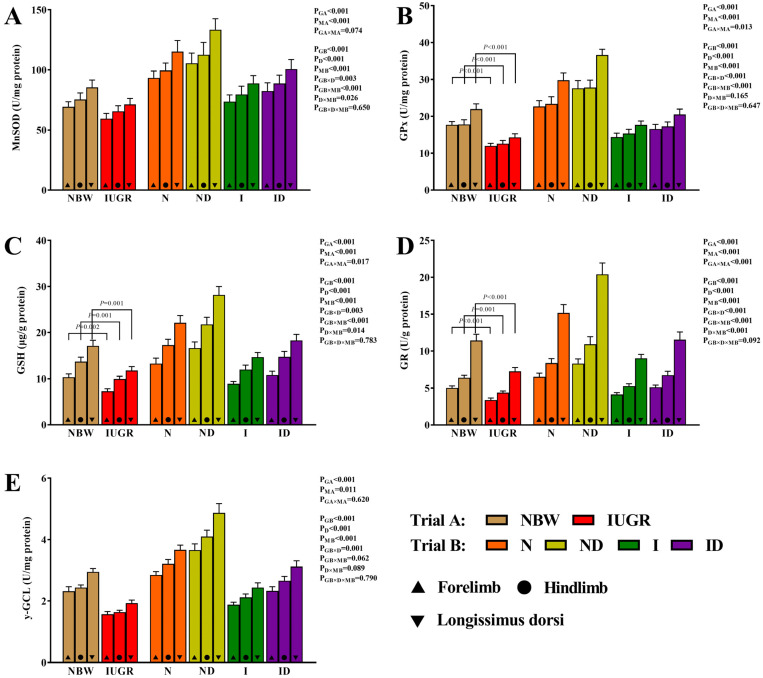
DMG-Na supplementation improved the mitochondrial redox status of the skeletal muscles of IUGR newborns during the suckling period. (**A**) MnSOD, (**B**) GPx, (**C**) GSH, (**D**) GR, and (**E**) γ-GCL values of the skeletal muscles. Data are shown as the mean ± standard deviation, n = 10 newborns per group. Different *p*-values lower than 0.05 represent significant differences. NBW, normal birth weight newborns; IUGR, intrauterine growth restriction newborns; N, NBW newborns are administered a sow milk diet; ND, NBW newborns are administered a sow milk diet + 0.1% DMG-Na; I, IUGR newborns are administered a sow milk diet; ID, IUGR newborns are administered a sow milk diet + 0.1% DMG-Na. MnSOD, manganese superoxide dismutase; GSH-Px, glutathione peroxidase; GSH, glutathione; GR, glutathione reductase; γ-GCL, γ-glutamylcysteine ligase.

Figure 4 illustrates the positive effects of DMG-Na on the mitochondrial redox status of the skeletal muscles of IUGR newborns during the suckling period. In trial A, the interaction effects (group × muscle) were significant for the mitochondrial GSH-Px (*p* = 0.013), GSH (*p* = 0.017), and GR (*p* < 0.001). The IUGR group’s mitochondrial GSH-Px, GSH, and GR levels were lower (*p* < 0.05) than those of the NBW group. In trial B, none of the assessed mitochondrial redox status traits were affected by the interaction effects (group × DMG-Na × muscle) that were significant (*p* > 0.05).

### 3.5. Oxidative Damage Study

**Figure 5:**
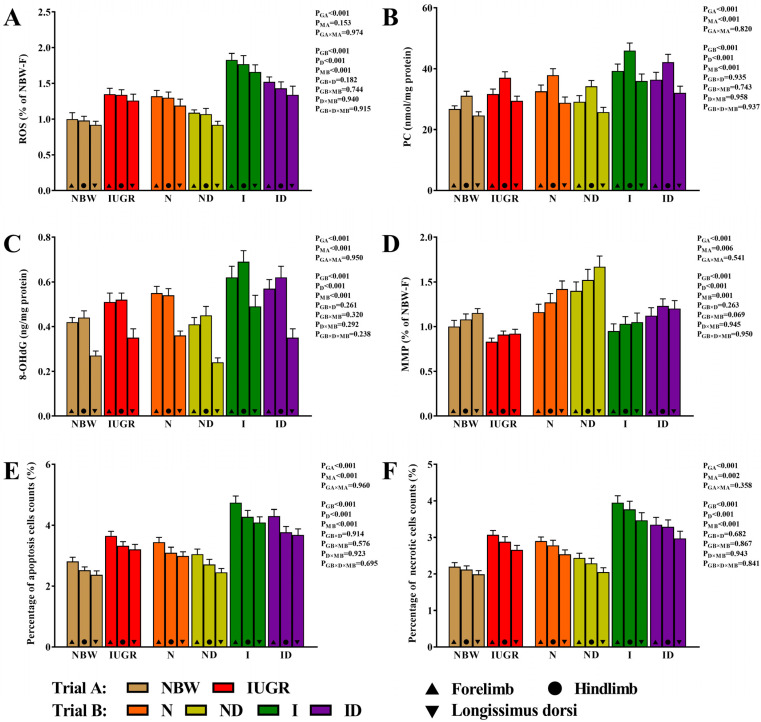
DMG-Na supplementation improved the oxidative damage of the skeletal muscles of IUGR newborns during the suckling period. (**A**) ROS, (**B**) PC, (**C**) 8-OHdG, (**D**) MMP, (**E**) percentage of apoptosis cells counts, and (**F**) percentage of necrotic cells counts values of the skeletal muscles. Data are shown as the mean ± standard deviation, n = 10 newborns per group. Different *p*-values lower than 0.05 represent significant differences. NBW, normal birth weight newborns; IUGR, intrauterine growth restriction newborns; N, NBW newborns are administered a sow milk diet; ND, NBW newborns are administered a sow milk diet + 0.1% DMG-Na; I, IUGR newborns are administered a sow milk diet; ID, IUGR newborns are administered a sow milk diet + 0.1% DMG-Na. ROS, reactive oxygen species; PC, protein carbonyls; 8-OHdG, 8-hydroxy-2-deoxyguanosine; MMP, mitochondrial membrane potential.

Figure 5 illustrates the protective effects of DMG-Na against oxidative damage to the skeletal muscles of IUGR newborns during the suckling period. For all of the assessed oxidative damage traits, the interaction impact in trial A (group × muscle) and the interaction effect in trial B (group × DMG-Na × muscle) were not significant (*p* > 0.05). The values of the skeletal muscles’ ROS, PC, 8-OHdG, MMP, apoptotic cell count (%), and necrotic cell count (%) were improved in the ND and ID groups than those in the N and I groups, respectively.

### 3.6. Mitochondrial ETC Complexes Study

**Figure 6:**
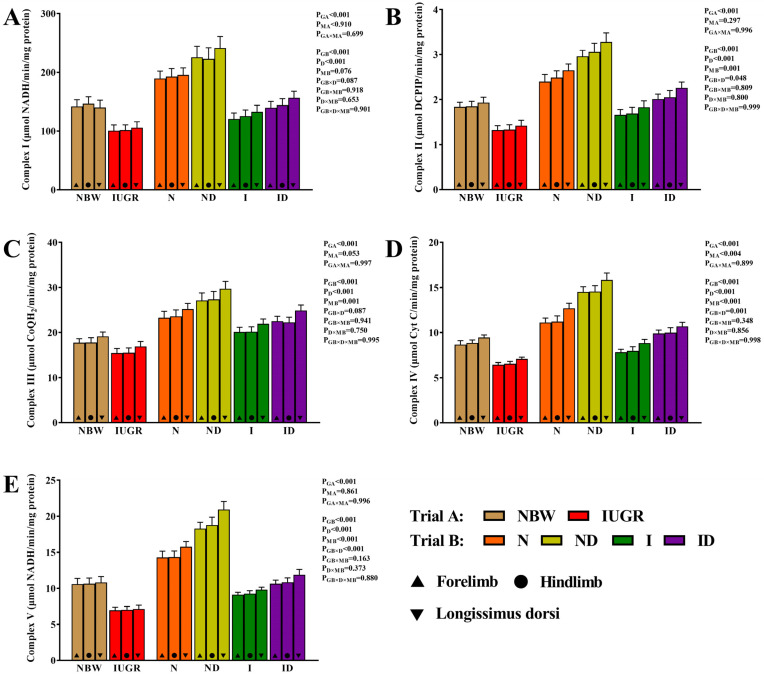
DMG-Na supplementation improved the mitochondrial ETC complexes of the skeletal muscles of IUGR newborns during the suckling period. (**A**) Complex I, (**B**) complex II, (**C**) complex III, (**D**) complex IV, and (**E**) complex V values of the skeletal muscles. Data are shown as the mean ± standard deviation, n = 10 newborns per group. Different *p*-values lower than 0.05 represent significant differences. NBW, normal birth weight newborns; IUGR, intrauterine growth restriction newborns; N, NBW newborns are administered a sow milk diet; ND, NBW newborns are administered a sow milk diet + 0.1% DMG-Na; I, IUGR newborns are administered a sow milk diet; ID, IUGR newborns are administered a sow milk diet + 0.1% DMG-Na.

Figure 6 presents the beneficial impacts of DMG-Na on the mitochondrial ETC complexes of the skeletal muscles of IUGR newborns throughout the suckling period. For all of the examined mitochondrial ETC complexes traits, neither the interaction effect in trial A (group × muscle) nor the interaction effect in trial B (group × DMG-Na × muscle) were significant (*p* > 0.05). However, the ND and ID groups improved levels of the mitochondrial ETC complexes I–V in the skeletal muscles than that of the N and I groups, respectively.

### 3.7. Energy Metabolism Study

**Figure 7:**
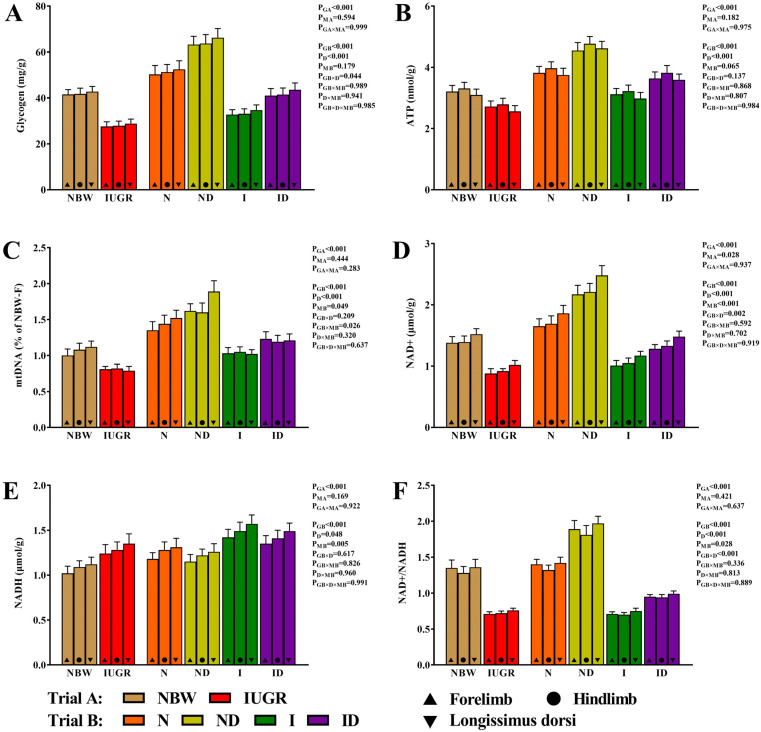
DMG-Na supplementation improved energy metabolism of the skeletal muscles of IUGR newborns during the suckling period. (**A**) glycogen, (**B**) ATP, (**C**) mtDNA, (**D**) NAD^+^, (**E**) NADH, and (**F**) NAD^+^/NADH values of the skeletal muscles. Data are shown as the mean ± standard deviation, n = 10 newborns per group. Different *p*-values lower than 0.05 represent significant differences. The mtDNA values of Forelinb in the NBW group were taken as a baseline and assigned as 1.0. NBW, normal birth weight newborns; IUGR, intrauterine growth restriction newborns; N, NBW newborns are administered a sow milk diet; ND, NBW newborns are administered a sow milk diet + 0.1% DMG-Na; I, IUGR newborns are administered a sow milk diet; ID, IUGR newborns are administered a sow milk diet + 0.1% DMG-Na. ATP, adenosine triphosphate; NAD^+^, nicotinamide adenine dinucleotide; NADH, nicotinamide adenine dinucleotide (reduced form).

The beneficial effects of DMG-Na on energy metabolism of the skeletal muscles of IUGR newborns during the suckling period are presented in Figure 7. For all of the examined energy metabolism features, neither the interaction effect in trial A (group × muscle) nor the interaction effect in trial B (group × DMG-Na × muscle) were significant (*p* > 0.05). The values of the skeletal muscles’ glycogen, ATP, mtDNA, NAD^+^, NADH, and NAD^+^/NADH were, however, improved in the ND and ID groups than those in the N and I groups, respectively.

### 3.8. Gene Expression Study

**Figure 8:**
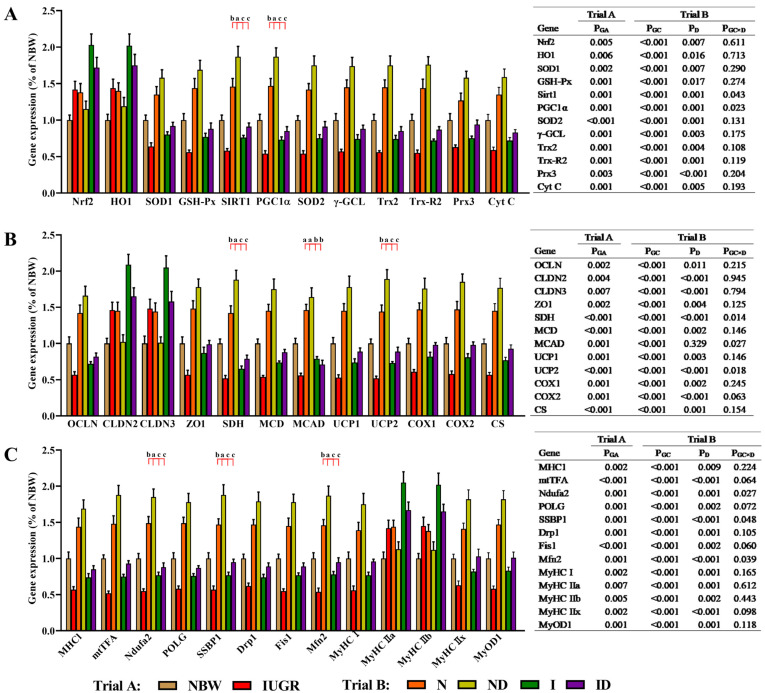
DMG-Na supplementation enhanced gene expression levels in the LM of IUGR newborns during the suckling period. (**A**) antioxidant-related, (**B)** cell adhesion-related and mitochondrial function-related, and (**C**) muscle growth-related gene expression levels of the LM. Data are shown as the mean ± standard deviation, n = 10 newborns per group. Different *p*-values lower than 0.05 represent significant differences and different superscripts a, b, c, d represent significant differences in Trial B (N, ND, I, and ID groups) (*p* < 0.05). NBW, normal birth weight newborns; IUGR, intrauterine growth restriction newborns; N, NBW newborns are administered a sow milk diet; ND, NBW newborns are administered a sow milk diet + 0.1% DMG-Na; I, IUGR newborns are administered a sow milk diet; ID, IUGR newborns are administered a sow milk diet + 0.1% DMG-Na. Nrf2, nuclear factor erythroid 2-related factor 2; HO1, heme oxygenase 1; SOD1, copper and zinc superoxide dismutase; GSH-Px, glutathione peroxidase; SIRT1, sirtuin 1; PGC-1α, peroxisome proliferator-activated receptor-γ coactivator-1α; SOD2, manganese superoxide dismutase; γ-GCL, γ-glutamylcysteine ligase; Trx2, thioredoxin 2; Trx-R2, thioredoxin reductase 2; Prx3, peroxiredoxin 3; Cyt C, Cytochrome C; OCLN, occludin; CLDN2, cloudin 2; CLDN3, cloudin 3; ZO1, zonula occludens-1; SDH, mitochondrial proteins succinate dehydrogenase; MCD, lipid oxidation enzymes malonyl-CoA decarboxylase; MCAD, medium-chain acyl-CoA dehydrogenase; UCP1, uncoupling protein 1; UCP2, uncoupling protein 2; COX1, cyclooxygenase 1; COX2, cyclooxygenase 2; CS, citrate synthase; MHC1, major histocompatibility complex I; mtTFA, mitochondrial transcription factor A; Ndufa2, NADH dehydrogenase (ubiquinone) iron-sulfur protein 2; POLG, γ DNA polymerases catalytic/accessory subunit; SSBP1, single-strand DNA binding protein 1; Drp1, dynamin-related protein 1; Fis1, mitochondrial fission 1; Mfn2, mitochondrial mitofusin 2; MyHC, myosin heavy chain; MyoD1, myogenic differentiation 1.

The beneficial effects of DMG-Na on mitochondrial function-related, cell adhesion-related, and muscle growth-related gene expressions in LM of IUGR newborns during the suckling period are shown in Figure 8. In trial A, all gene expressions in LM of IUGR newborns were deficient (*p* < 0.05) compared to those in LM of NBW newborns. In trial B, the interaction effects (group × DMG-Na) were significant for SIRT1 (*p* = 0.043), PGC-1α (*p* = 0.023), SDH (*p* = 0.014), MCAD (*p* = 0.027), UCP2 (*p* = 0.018), Ndufa2 (*p* = 0.027), SSBP1 (*p* = 0.048), and Mfn2 (*p* = 0.039). As compared to the N and I groups, these gene expressions in LM were increased (*p* < 0.05) in the ND and ID groups, respectively.

### 3.9. Western Blot Study

**Figure 9:**
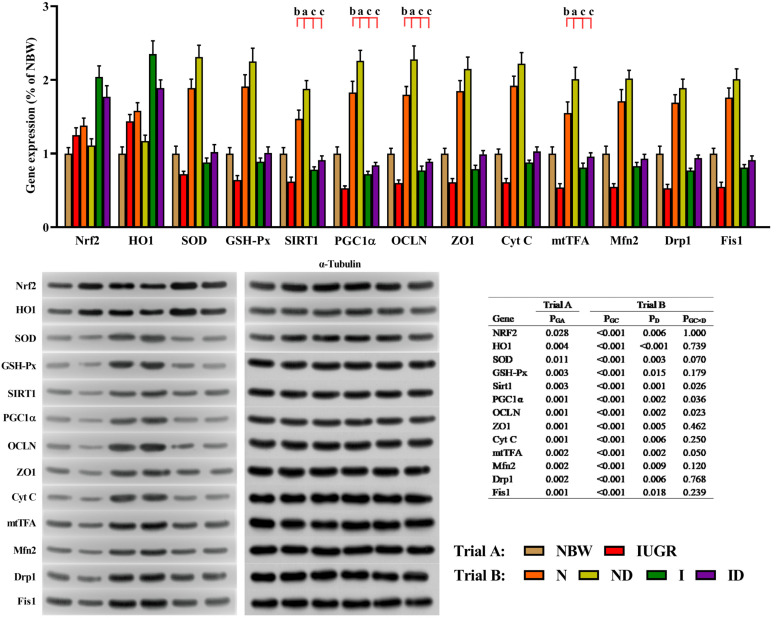
DMG-Na supplementation enhanced protein expression levels in the LM of IUGR newborns during the suckling period. Data are shown as the mean ± standard deviation, n = 10 newborns per group. Different *p*-values lower than 0.05 represent significant differences and different superscripts a, b, c, d represent significant differences in Trial B (N, ND, I, and ID groups) (*p* < 0.05). NBW, normal birth weight newborns; IUGR, intrauterine growth restriction newborns; N, NBW newborns are administered a sow milk diet; ND, NBW newborns are administered a sow milk diet + 0.1% DMG-Na; I, IUGR newborns are administered a sow milk diet; ID, IUGR newborns are administered a sow milk diet + 0.1% DMG-Na. Nrf2, nuclear factor erythroid 2-related factor 2; HO1, heme oxygenase 1; SOD, superoxide dismutase; GSH-Px, glutathione peroxidase; Sirt1, sirtuin 1; PGC1α, peroxisome proliferator-activated receptorγcoactivator-1α; OCLN, occluding; ZO1, zonula occludens-1; CytC, Cytochrome C; mtTFA, mitochondrial transcription factor A; Mfn2, mitochondrial mitofusin2; Drp1, dynamin-related protein 1; Fis1, mitochondrial fission 1.

The beneficial effects of DMG-Na on mitochondrial function-related and cell adhesion-related protein expression in LM of IUGR newborns during the suckling period are shown in Figure 9. In trial A, all gene expressions in LM of IUGR newborns were deficient (*p* < 0.05) compared to those in LM of NBW newborns. In trial B, the interaction effects (group × DMG-Na) were significant for SIRT1 (*p* = 0.026), PGC-1α (*p* = 0.036), OCLN (*p* = 0.023), and mtTFA (*p* = 0.050). In the ND and ID groups, as opposed to the N and I groups, the expression of these proteins in LM was improved (*p* < 0.05).

## 4. Discussion

### 4.1. The Reduced Performance of IUGR Newborns Was Reversed by DMG-Na Supplementation

Since it hinders postnatal development, results in permanent oxidative damage, and compromises the health of skeletal muscles, IUGR is a significant issue in human medicine [15]. IUGR newborn piglet models have been utilized in certain research to demonstrate newborns’ poor performance [1,2,16], and these findings support the current study’s choosing of the NBW newborns and IUGR newborns, who had birth weights of 1.53 ± 0.04 kg and 0.76 ± 0.06 kg, respectively. Since skeletal muscle makes up the majority of the total body mass and is the principal organ responsible for maintaining metabolic homeostasis, it is particularly vulnerable to oxidative damage. In this study, supplementing with DMG-Na during the suckling period increased the growth performance of IUGR newborns relative to NBW newborns; nevertheless, more research is needed to pinpoint the precise mechanism.

### 4.2. The Reduced Redox Status of IUGR Newborns Was Improved by DMG-Na Supplementation

Oxidative damage can raise the amount of ROS, which can affect the redox status, damage mitochondrial structure, and impair mitochondrial function [17]. SOD, which catalyzes the proportional conversion of endogenous superoxide anions to hydrogen peroxide and is ultimately neutralized by intracellular CAT and GSH-Px, might help with this. MnSOD, GSH, GR, and γ-GCL are essential for preventing mitochondrial oxidative damage. Prior research suggested that DMG-Na might function as an antioxidant addition to enhance the body’s redox status and mitigate the oxidative damage brought on by excessive ROS formation [10,11,12]. By scavenging excessively produced ROS in this study, DMG-Na demonstrated positive effects on the redox status and preserved the equilibrium of the intracellular redox status, which is expected to result in favorable histological examination results.

The redox status system, which is altered by oxidative stress, maintains a dynamic equilibrium with the ROS level in the cells [18]. As a result of structural damage and DNA and mitochondrial malfunction brought on by excessive ROS production, the redox status is eventually impacted [19]. IUGR is intimately linked to metabolic syndrome, mitochondrial dysfunction, oxidative damage, and elevated ROS levels. According to some studies, elevated ROS levels cause mtDNA damage, whereas decreased mitochondrial function increases endogenous ROS levels. MMP level, which has a negative correlation with ROS concentration, serves as a sign that mitochondria-dependent apoptosis is starting. Another study discovered that newborns with IUGR have a decreased redox status and are more vulnerable to oxidative damage [20]. Another study discovered that newborns with IUGR have a decreased redox status and are more vulnerable to oxidative damage [1]. In the current study, a decrease in redox status in newborns with IUGR consistently resulted in decreased mitochondrial activity in the skeletal muscle. A number of studies have confirmed that natural antioxidants may shield cells from oxidative damage, and DMG-Na can also mitigate the oxidative damage brought on by excessive ROS formation [10,11,12]. Further research is needed to determine how DMG-Na affects skeletal muscle oxidative damage reduction.

### 4.3. The Mitochondrial Dysfunction of IUGR Newborns Was Ameliorated by DMG-Na Supplementation via the Nrf2/SIRT1/PGC-1α Network

The mtDNA copy number, required for growth and glycogen production and related to mitochondrial number, may be used to quantitatively quantify ATP. Newborns with IUGR had reduced ATP levels, which affected postnatal muscle development and glycogen storage [10]. It is possible that the loss of complex I activity brought on by oxidative damage to the skeletal muscle mitochondria of newborns with IUGR will reduce the production of mitochondrial energy. According to this study, the decreased activity of complex I, which couples electrons from NADH to quinone and transports a proton across the inner mitochondrial membrane to produce ATP, might account for the low NAD^+^/NADH ratio. Additionally, it prevented the mitochondrial transit of glycolytic and tricarboxylic acid cyclic metabolites, resulting in a decrease in the production of ATP [21]. Bioenergetics, which employs the exergonic proton backflow for ATP synthesis from ADP and inorganic phosphate in the matrix, depends on an increase in complex V or ATP synthase activity in the skeletal muscle mitochondria of IUGR newborns during the suckling period [22]. The skeletal muscles are protected from oxidative damage and are maintained in their normal structure and function by DMG-Na, as shown in a previous study [10]. Another study discovered that DMG-Na had a positive impact on cells, shielding them from oxidative damage [11,12], and this finding may be one of the factors contributing to the ID group’s improved skeletal muscle mitochondrial ETC complex function and energy metabolism.

By regulating the expression of genes linked to redox status (SOD, GSH-Px, and γ-GCL), Nrf2 and HO1 activation is crucial for reducing oxidative damage. Trx2, Trx-R2, and Prx3 proteins are abundant in mitochondria and work in concert to avoid oxidative damage by scavenging several free radicals and controlling mitochondria-dependent apoptotic pathways [6]. PGC-1α is a pleiotropic coactivator that regulates mitochondrial biogenesis (COX1, Cyt C, MHC1, mtTFA, Ndufa2, and UCP1) as well as mitochondrial function-related gene expression [mtDNA replication and repair (POLG1, POLG2, and SSBP1), mitochondrial fission (Drp1 and Fis1), and mitochondrial fusion (Mfn2)], as it induces mitochondrial gene expression at the level of nuclear and mitochondrial genomes [7]. Due to its sensitivity to cellular redox and nutritional condition, SIRT1 was first identified as a factor controlling apoptosis and DNA repair. It is also known to regulate genomic stability and cellular metabolism [8]. According to previous studies, SIRT1 physically interacts with and deacetylates PGC-1α at a number of lysine sites, which increases PGC-1α activity and controls the redox status, lipid oxidation enzymes (MCD and MCAD), and mitochondrial gene expression (SDH, UCP2, COX2, and CS) [9]. Key regulators of cell permeability include OCLN, CLDN, and ZO1, which is linked with paracellular permeability [23]. Meat quality is monitored by the expression of myosin heavy chain (MyHC) family, among which MyHC IIb expression level is negatively correlated with the meat quality. Myogenic differentiation 1 (MyoD1) can transform various types of cells into myoblasts and promote their differentiation into mature muscle fibers [24]. It is one of the key regulatory factors that controls the growth of skeletal muscles. To the best of knowledge, this is the first study to demonstrate the benefits of DMG-Na on the LM redox status and mitochondrial function of IUGR newborns during the suckling period via the Nrf2/SIRT1/PGC-1α network, and additional research is required to clarify this particular mechanism.

## 5. Conclusions

In conclusion, our study showed that DMG-Na might significantly lessen skeletal muscle structural damage and dysfunction in newborns with IUGR throughout the suckling period. In addition, we found that DMG-Na may, via the Nrf2/SIRT1/PGC-1α network, directly neutralize excessive free radicals, indirectly enhance redox status, and prevent the aberrant expression of stress-related genes. As a result, these results imply that DMG-Na may perform as a drug that promotes health and may be utilized to treat skeletal muscle abnormalities in newborns with IUGR in order to enhance their performance throughout the suckling period.

## Figures and Tables

**Table 1:** DMG-Na supplementation improved the body weight of IUGR newborns during the suckling period ^1^.

	Treatment ^2^				*p* Value						
Item	N	ND	I	ID	P_G_	P_T_	P_D_	P _G×T_	P _G×D_	P _T×D_	P _G×T×D_
0 d	1.53 ± 0.24 ^a^	-	0.76 ± 0.10 ^b^	-	<0.001	<0.001	<0.001	0.004	0.021	0.002	<0.001
7 d	3.48 ± 0.26 ^a^	-	2.23 ± 0.21 ^b^	-							
10 d	3.99 ± 0.35 ^b^	5.03 ± 0.31 ^a^	2.88 ± 0.31 ^c^	3.24 ± 0.35 ^bc^							
13 d	5.02 ± 0.37 ^b^	6.51 ± 0.36 ^a^	3.48 ± 0.36 ^c^	4.03 ± 0.37 ^c^							
16 d	6.31 ± 0.46 ^b^	7.45 ± 0.36 ^a^	4.35 ± 0.46 ^c^	5.08 ± 0.45 ^b^							
19 d	7.01 ± 0.48 ^b^	8.12 ± 0.42 ^a^	5.00 ± 0.50 ^c^	6.15 ± 0.43 ^b^							
21 d	7.71 ± 0.58 ^b^	8.90 ± 0.52 ^a^	5.88 ± 0.60 ^c^	7.07 ± 0.57 ^b^							

^1^ Data are shown as the mean ± standard deviation, n = 10 newborns per group. Significant differences are indicated by various superscripts a, b, and c (N, ND, I, ID group) (*p* < 0.05). ^2^ NBW, normal birth weight newborns; IUGR, intrauterine growth restriction newborns; N, NBW newborns are administered a sow milk diet; ND, NBW newborns are administered a sow milk diet + 0.1% DMG-Na; I, IUGR newborns are administered a sow milk diet; ID, IUGR newborns are administered a sow milk diet + 0.1% DMG-Na.

## Data Availability

Not applicable.

## Appendix A

**Table A1:** Composition and nutrient level of the sow diet in gestation and lactation feeds.

	Gestation Phase	Lactation Phase
Ingredients		
Corn (%)	80	66
Soybean meal (%)	16	28
Soybean oil (%)		2
Premix (%) ^a^	4	
Premix (%) ^b^		4
Calculated nutritional values		
Metabolizable energy (kcal/kg)	3176.64	3259.04
Protein (%)	13.86	18.24
Crude fibre (%)	2.25	2.66
Digestible lysine (%)	0.64	1.03
Digestible methionine (%)	0.24	0.29
Digestible threonine (%)	0.54	0.71
Calcium (%)	0.71	0.57
Phosphorus (%)	0.39	0.41
Sodium (%)	0.18	0.18

^a^ Content per kilogram of product: chlorine 0.29%; copper 32.76 mg/kg; iron 180.08 mg/kg; manganese 50.50 mg/kg; selenium 0.13 mg/kg; zinc 127.14 mg/kg; cobalt 2.00 mg/kg; iodine 6.32 mg/kg; organic manganese 20.00 mg/kg; organic iron 50.00 mg/kg; organic copper 10.00 mg/kg; organic chromium 400.00 mg/kg; phytase HiPhos M 2000.00 FYT. ^b^ Content per kilogram of product: chlorine 0.29%; copper 28.03 mg/kg; iron 195.22 mg/kg; manganese 53.72 mg/kg; selenium 0.57 mg/kg; zinc 131.30 mg/kg; cobalt 1.00 mg/kg; iodine 1.03 mg/kg; organic manganese 20.00 mg/kg; organic iron 2.00 mg/kg; organic copper 0.40 mg/kg; organic chromium 16.00 mg/kg; phytase HiPhos M 2000.00 FYT.

**Table A2:** Primer sequences used for Real-time PCR assay.

Gene ^1^	Primer Sequence ^2^	Genbank ID ^3^	Gene ^1^	Primer Sequence ^2^	Genbank ID ^3^
*β-actin*	Forward: GTTCGAGACCTTCAACACGC	XM_003357928.3	*MCAD*	Forward: AAACCAGACCTTCGGTAGCA	NM_214039.1
	Reverse: CCATGACAATGCCAGTGGTG			Reverse: GTATTTCGGCGACCAGAATC	
*Nrf2*	Forward: GACAAACCGCCTCAACTCAG	NM_001114671.1	*UCP1*	Forward: AAGCAAGGAGCATTCCTGGG	XM_013978885.1
	Reverse: GTCTCCACGTCGTAGCGTTC			Reverse: CTGCCCTGTGGGTAACCATT	
*HO1*	Forward: ACACTGGGCTGGAGAGATCA	NM_001138825.2	*UCP2*	Forward: CGTGGTAAAGGTCCGGTTC	NM_214289.1
	Reverse: AGAGTAGCGTATGTGGTGCC			Reverse: AGGAGCGTGTCCTTGATGAG	
*SOD1*	Forward: AGGGAGAGAAGACAGTGTTAGT	NM_001190422.1	*COX1*	Forward: GCAGTTGCCAGATGCTGAAC	XM_001926129.5
	Reverse: GTACACAGTGGCCACACCAT			Reverse: TGGGTGAAGTGTTGGGCAAA	
*GSH-Px*	Forward: CTTATGGTGGGGTAGGGGGA	XM_003463071.2	*COX2*	Forward: GAGACAGCATAAACTGCGCC	NM_214321.1
	Reverse: GGCTGGGTAGGAGATTGCAG			Reverse: AGTGTCTTTGGCTGTCGGAG	
*Sirt1*	Forward: GGTGGTTCCTCGATGTCCTA	NM_001145750.1	*CS*	Forward: CCACAGTGACCATGAAGGTG	NM_214276.1
	Reverse: GGTGAGGCAAAGGTTCCCTA			Reverse: AACCCGTCCTGAGTTGAGTG	
*PGC1α*	Forward: GGGGCCCATGGGAATCATC	XM_013992150.1	*MHCI*	Forward: GAAGCCTTCTGGACACCTTCA	AF013960.1
	Reverse: AACTGCTGTTGTTTGGGCCT			Reverse: CCAGAACCAGGCCAACAGT	
*SOD2*	Forward: GGCGCCATCAAGTTCAACAG	NM_001281206.1	*mtTFA*	Forward: TATAGGGCAGACTGGCAGGT	NM_001130211.1
	Reverse: TGAGCCTTTTGGGGGTTCAC			Reverse: TGGACCATCCTTAGCTTCCT	
*γ-GCL*	Forward: CCCTTGTGGTACCTCTGCAT	XM_013977739.1	*Ndufa2*	Forward: TGCTAAGTGGCAAAGCCTGA	XM_003124046.3
	Reverse: TGGGTTTGTCCTTTCCCCCT			Reverse: GAACAAAACATCCGGAGGCG	
*Trx2*	Forward: GGACCGAATCTGAGCCAAGT	NM_123358.4	*POLG*	Forward: AGCAGAAGCCCCAAAGTTCC	XM_001927064.4
	Reverse: GCACCATGAGGCCGAGAAAT			Reverse: AGCATGACCTCTCTCCCTTCT	
*Trx-R2*	Forward: GATACCCCACGCACATCGAA	NM_001282512.1	*SSBP1*	Forward: CATACCAAATGGGCGATGTC	XM_013985577.1
	Reverse: CCCGACCACCAACGTTTTTC			Reverse: TTGTGGTTGCTTGTCGTCTC	
*Prx3*	Forward: CAATGTCGACCGCAAGAACG	NM_079663.3	*Drp1*	Forward: AAGAGGAGTCGCATGTGTCG	XM_001928848
	Reverse: GTCAATGATGAAGGTGCCGC			Reverse: TGCAATCATGCCCACCAGAT	
*Cyt C*	Forward: TCGGAGTCACCAGTGCTAGA	XM_003127002.3	*Fis1*	Forward: AGTAGTGAGGATTGCGAGGC	XM_021086263
	Reverse: GGAACATTGAGGCCTACGGA			Reverse: TACTTGCTTCGCACCAGACA	
*OCLN*	Forward: CCTCCTCCCCTTTCGGACTA	NM_001163647	*Mfn2*	Forward: TAGTAGGGTCGAACACGCTG	XM_021095371
	Reverse: TAGACCCCTAGCCTGGGAAC			Reverse: GATGCCCCTCACTTTGGACA	
*CLDN2*	Forward: ATCAAGCAAGGGCAGAAACG	XM_021079578	MyHC I	Forward: CGTGGACTACAACATCATAGGC	NM_213855
	Reverse: GGATGCGATTGGTGGGTTTG			Reverse: CCTTCTCAACAGGTGTGTCG	
*CLDN3*	Forward: TGTCCGTCTATCCGTCCGTC	NM_001160075	MyHC IIa	Forward: CATTGAGGCCCAGAATAGGC	NM_214136.1
	Reverse: ATCCGCGCTGTGATAATGCT			Reverse: TGCTTCCGTCTTCACTGTCAC	
*ZO1*	Forward: CTGCCAAGTGAAACTGCACA	AJ318101	MyHC IIb	Forward: GACTCTGGCTTTCCTCTTTGC	NM_001123141.1
	Reverse: GACAGAGAACGTGTCAACGC			Reverse: GAGCTGACACGGTCTGGAAA	
*SDH*	Forward: ATGGAAAACGGGGAGTGTCG	CX063991.1	MyHC IIx	Forward: TTGACTGGGCTGCCATCAAT	NM_001104951.2
	Reverse: TTCCGGTAGCGACAACAGTG			Reverse: GCCTCAATGCGCTCCTTTTC	
*MCD*	Forward: ACAGATGTGAGGCTCTGTGC	FJ263687	MyOD1	Forward: TTCCGACGGCATGATGGATT	NM_001002824.1
	Reverse: CTCATGTCTGGATCTCCGGC			Reverse: GCTGTAATAGGTGCCGTCGT	

^1^ Nrf2, nuclear factor erythroid 2-related factor 2; HO1, heme oxygenase 1; SOD1, copper and zinc superoxide dismutase; GSH-Px, glutathione peroxidase; SIRT1, sirtuin 1; PGC-1α, peroxisome proliferator-activated receptor-γ coactivator-1α; SOD2, manganese superoxide dismutase; γ-GCL, γ-glutamylcysteine ligase; Trx2, thioredoxin 2; Trx-R2, thioredoxin reductase 2; Prx3, peroxiredoxin 3; Cyt C, Cytochrome C; OCLN, occludin; CLDN2, cloudin 2; CLDN3, cloudin 3; ZO1, zonula occludens-1; SDH, mitochondrial proteins succinate dehydrogenase; MCD, lipid oxidation enzymes malonyl-CoA decarboxylase; MCAD, medium-chain acyl-CoA dehydrogenase; UCP1, uncoupling protein 1; UCP2, uncoupling protein 2; COX1, cyclooxygenase 1; COX2, cyclooxygenase 2; CS, citrate synthase; MHC1, major histocompatibility complex I; mtTFA, mitochondrial transcription factor A; Ndufa2, NADH dehydrogenase (ubiquinone) iron-sulfur protein 2; POLG, γ DNA polymerases catalytic/accessory subunit; SSBP1, single-strand DNA binding protein 1; Drp1, dynamin-related protein 1; Fis1, mitochondrial fission 1; Mfn2, mitochondrial mitofusin 2; MyHC, myosin heavy chain; MyoD1, myogenic differentiation 1. ^2^ Shown as forward primer followed by reverse primer. ^3^ GenBank Accession Number.
